# Definitions of “rural” and “urban” and understandings of economic transformation: Evidence from Tanzania

**DOI:** 10.1016/j.jrurstud.2020.08.014

**Published:** 2020-10

**Authors:** Ayala Wineman, Didier Yélognissè Alia, C. Leigh Anderson

**Affiliations:** Daniel J. Evans School of Public Policy and Governance, University of Washington, Parrington Hall, 4100 15th Ave NE, Seattle, WA, 98195, USA

**Keywords:** Big data, Measurement, Remotely sensed data, Rural transformation, Tanzania, Urban

## Abstract

Designing effective policies for economic development often entails categorizing populations by their rural or urban status. Yet there exists no universal definition of what constitutes an “urban” area, and countries alternately apply criteria related to settlement size, population density, or economic advancement. In this study, we explore the implications of applying different urban definitions, focusing on Tanzania for illustrative purposes. Toward this end, we refer to nationally representative household survey data from Tanzania, collected in 2008 and 2014, and categorize households as urban or rural using seven distinct definitions. These are based on official administrative categorizations, population densities, daytime and nighttime satellite imagery, local economic characteristics, and subjective assessments of Google Earth images. These definitions are then applied in some common analyses of demographic and economic change. We find that these urban definitions produce different levels of urbanization. Thus, Tanzania's urban population share based on administrative designations was 28% in 2014, though this varies from 12% to 39% with alternative urban definitions. Some indicators of economic development, such as the level of rural poverty or the rate of rural electrification, also shift markedly when measured with different urban definitions. The periodic (official) recategorization of places as rural or urban, as occurs with the decennial census, results in a slower rate of rural poverty decline than would be measured with time-constant boundaries delimiting rural Tanzania. Because the outcomes of analysis are sensitive to the urban definitions used, policy makers should give attention to the definitions that underpin any statistics used in their decision making.

## Highlights

• The choice of rural definition can affect our understanding of economic change.
• We use administrative, remotely sensed, and survey-based data in Tanzania.
• Some indicators of economic development shift with different rural definitions.
• Different definitions tend to paint a consistent picture of rural farms.
• Periodic rural/urban recategorization affects the pace of rural poverty decline.

## 1 Introduction

Designing effective policies for economic development and sustainable rural transformation, and monitoring progress toward the associated policy goals, often entails categorizing populations by their rural or urban status. Yet there exists no universal definition of what constitutes an “urban” area; countries alternately apply criteria related to settlement size, population density, or economic advancement (UN, 2018; Potts, 2017; Lazaro et al., 2017). Some definitions may overlook the emergence of new urban spaces within previously rural landscapes, with potentially deleterious effects for resource allocation (Fox et al., 2018; Ingelaere et al., 2018; Lazaro et al., 2017; Muzzini and Lindeboom, 2008). Other definitions may conflate density with urbanity, even where economic transformation has not occurred (Potts, 2018).

Why does the definition used to classify areas as rural or urban matter? First, the choice of urban definition can affect our understanding of the level and rate of urbanization in a country. These metrics are tracked closely as indicators of economic development and can affect decisions around the relative priority given to rural and urban areas (Potts, 2018). This may be particularly relevant for national budget allocations to ministries that focus on either rural or urban populations. Second, the choice of urban definition can affect measures of urban and rural poverty and our understanding of how rural areas are transformed in the course of economic development (for example, agriculture's declining dominance in the rural economy). Third, the application of different definitions in different countries can confound cross-country analyses, particularly with respect to comparisons of urbanization levels (Satterthwaite, 2010; Cohen, 2004).

In this study, we draw from a wide set of data sources (administrative, remotely sensed, and survey-based) to explore the implications of applying different urban definitions, and we focus on Tanzania for purposes of illustration. We specifically ask the following research questions:

1. How do different urban definitions affect the estimated level and pace of urbanization in Tanzania?
2. How do different urban definitions affect comparisons of urbanization across Tanzania and another country, namely Nigeria?
3. How does the application of different urban definitions in Tanzania affect measures of welfare and economic orientation in the rural population? Examples include the rural poverty rate and households' agricultural orientation.
4. How does this affect measures of agricultural indicators within the rural farming population? An example is the rate at which farmers market their agricultural production.
5. How does the recategorization of places as rural or urban over time affect the rate at which rural welfare is found to have improved or deteriorated in Tanzania?

This paper makes several contributions to the literature. First, many studies rely only on official (nationally defined) definitions of rural and urban when evaluating processes of change in rural areas. In this paper, we carefully document how the measured rates of urbanization and any trends evident in rural economies vary when analysts draw from alternative (and often newly available) data sources to delineate what is “rural”. To our knowledge, no other study incorporates such a wide range of data sources to identify urban spaces. Second, international statistical databases, such as the World Bank's World Development Indicators, similarly rely on official definitions. In this paper, we highlight the manner in which different urban definitions can lead to diverging conclusions, including when comparing statistics across countries. Third, we consider the implications of measuring rural poverty over time when the official boundaries around what is “rural” are periodically redrawn. To our knowledge, no other study engages with this question in an African setting. Fourth, we supplement the paper with detailed documentation of our methods. We hope this will be useful to analysts who wish to utilize secondary data sources but do not realize these are so readily accessible.

The remainder of the paper is structured as follows. A review of the literature on structural and rural transformation and the relevance of urban definitions is provided in Section 2. Section 3 contains an introduction to the data sources used in analysis, and Section 4 delineates the definitions of “urban” that will be applied and discusses the strengths and limitations of each. Results are presented in Section 5, and Section 6 concludes with a discussion of the results and their implications. Detailed documentation of our methods is available in appendix.

## 2 Background

### 2.1 Urbanization and urban definitions

Urbanization can be defined as the shift from a dispersed population toward one that increasingly resides in densely populated settlements in which non-agricultural economic activities are dominant (Cockx et al., 2018; Fox et al., 2018). There are three main sources (causes) of urbanization. First, a natural increase in the urban population occurs when there are more births than deaths in urban areas, and the urban share of a country's population grows when the ratio of births to deaths in urban areas exceeds that of rural areas (Jedwab et al., 2018). Second, urbanization occurs with migration when more people move from rural to urban areas than in the reverse direction. Third, urbanization can occur with reclassification in which previously rural territories are newly classified as urban. The latter may take the form of the spatial expansion of urban areas (i.e., the annexation of spaces around a city's periphery). Alternatively, in the process of rural transformation, rural settlements may “graduate” into urban status when their populations exceed some threshold (Muzzini and Lindeboom, 2008; Cohen, 2004; Bloom et al., 2010).

Many countries base their urban definition on settlement (population) size, applying a certain threshold to distinguish between urban and rural areas. Yet there remain ambiguities as to the placement of settlement boundaries or what threshold should be applied, and the choice of threshold can affect a country's measured urbanization level (Potts, 2018; Satterthwaite, 2010). In many countries, settlements of a few thousand people tend to include shops, services, and manufacturing, while elsewhere, even larger settlements tend to be centered around farming (Satterthwaite, 2006). Countries may also apply an urban definition based on population density, which is recognized as a defining feature of urban centers. Of 231 countries or areas captured in the 2011 World Urbanization Prospects (UN, 2012), 107 incorporate settlement size or population density in their urban definitions, and these are the sole criteria used in 48 countries.

Economic characteristics related to urban function, such as the absence of agricultural land or employment, sometimes factor into urban definitions (Potts, 2013). For example, Zambia classifies as urban all localities with at least 5000 inhabitants and a majority of the labor force not engaged in agricultural activities (Bloom et al., 2010). As of 2011, economic criteria were included in the urban definitions of 33 countries or areas, and characteristics such as the existence of paved streets, water systems, or sewage systems were factored into the urban definition in 43 cases (UN, 2012). Over half of all countries reporting to the UN cite administrative criteria in their urban definition without reporting a more detailed (quantitative) basis for the designation (Potts, 2017; UN, 2012).

Some urban definitions may overlook the existence of urban spaces within rural landscapes. Growing villages often attract migrants from more sparsely populated areas and become hubs for employment, trade, and services (Lazaro et al., 2017). These sites are alternately referred to as “village towns”, “emerging urban centers”, or “intermediate urban centers”, and they have considerable economic importance (Lazaro et al., 2017; Satterthwaite, 2006). In the Kagera region of Tanzania, more than twice as many people are found to move from rural villages to towns/small cities than to large cities (defined as having populations of at least one-half million) (Christiaensen et al., 2013). For this reason, in aggregate, migration to small urban centers reduces poverty more than migration to larger cities.

These small urban centers have mushroomed in Tanzania, increasing in number from 150 to 600 between 2002 and 2012 (Lazaro et al., 2017). According to Satterthwaite (2006), despite the importance of these newly urban settings, they tend not to be recognized as urban by rural specialists—even within the discourse on non-farm employment. This oversight has potential consequences. If urbanization is occurring off the radar of government agencies, policies cannot be designed to address the challenges associated with this type of urbanization. Government structures suitable for villages may be unable to oversee settlements that are genuinely (though not officially recognized as) urban (Muzzini and Lindeboom, 2008), and needs for infrastructure, housing, and health, education, and financial services may be unmet (Lazaro et al., 2017).

Other definitions of urban centers may conflate population density with urbanity, even where economic transformation towards industry has not occurred. As noted earlier, many countries consider population density within their urban definitions. However, high rural population densities can be supported by favorable growing conditions, and in Kenya, Potts (2017) argues that the use of population density criteria in some urban definitions leads to the misclassification of many rural people whose livelihoods still revolve around agriculture. This can be problematic, particularly if data on the urban population are understood by policy makers to be indicators of structural economic transformation.

### 2.2 Structural, rural, and agricultural transformation

The definition used to categorize populations as urban or rural can influence our understanding of the urban transition and structural transformation in the course of economic development. In the process of structural transformation, predominantly agricultural societies transition to become higher-income societies with a focus on the higher-return nonfarm sector, and this shift should manifest not only in the physical concentration of the population in urban centers, but also in the gradual shift in rural areas away from an agricultural focus and towards a more diversified economy (Barrett et al. 2017a, 2017b; Davis et al., 2017; Jayne et al., 2018). Thus, the process of structural transformation encompasses changes within the rural economy and the agricultural sector. In “rural push” theories of structural transformation, agricultural transformation is understood to catalyze wider transformation by releasing labor to nonfarm economic activities (Johnston and Mellor, 1961). Such agricultural transformation is characterized by the adoption of modern farm inputs and practices, improved agricultural productivity, improved access to output markets, and a more commercial orientation of farms (Timmer, 1988). With rising incomes, farms exhibit multiplier effects in the rural nonfarm economy by demanding more goods and services; in turn, the strengthened nonfarm economy further encourages workers to exit farming (Jayne et al., 2018; Johnston and Mellor, 1961). As farms increasingly send supplies to the market, better functioning food markets also enable more people to pivot away from agriculture by ensuring they will still have reliable food access.

Structural transformation is inherently a story of income growth, and therefore the poverty rate in both urban and rural areas should fall in the process. Other indicators of rural welfare, including education and access to amenities, are also expected to improve. Following Engel's Law, as incomes rise, the share of income spent on food falls, and as the rural population shifts away from agriculture, the share of food that is accessed through purchase rather than home production is expected to rise. A country's trajectory along the arc of structural transformation can therefore be tracked by measuring the level of urbanization, the economic orientation of the rural economy, and the rate of farm commercialization in rural areas, among other factors. However, this task necessarily requires a definition of what is considered “rural” versus “urban”.^1^

This study focuses on Tanzania for illustrative purposes, although we expect the exercise to also be relevant in other low-income countries. Tanzania seems to be growing increasingly urbanized over the 2008–2014 time period (Wineman et al., 2020), although note that this assertion necessarily rests on the official (administrative) definition of “urban” in this setting. Tanzania seems to also be undergoing other changes consistent with structural and rural transformation. Nationwide, poverty rates are falling and household income portfolios are monotonically shifting away from agriculture in favor of non-agricultural wage work or self-employment. Agricultural households seem to be increasingly engaging with markets for factors of agricultural production (land and labor) and also to adopt some modern technologies and exhibit a commercial (rather than subsistence) orientation for their farms. As food markets grow more reliable in Tanzania, we further observe that households increasingly procure food through the market rather than home production (ibid). In this study, we will look closely at these documented patterns to understand whether they are sensitive to the urban definition being applied.

## 3 Data sources and key variables

This study draws primarily on the Living Standards Measurement Study – Integrated Surveys on Agriculture (LSMS-ISA) in Tanzania.^2^ This nationally representative household survey, implemented by the Tanzania National Bureau of Statistics, was administered in 2008/09, 2010/11, 2012/13, and 2014/15, and we draw from the first and most recent waves in this analysis. The Tanzania LSMS-ISA sample is stratified to capture both urban and rural areas within each region of the country (with the exception of the Dar es Salaam region, which is entirely urban). In addition to being nationally representative, this data set allows for analysis of four strata within the country, including Dar es Salaam, other urban areas on mainland Tanzania, rural mainland Tanzania, and Zanzibar (Tanzania NBS 2016). The sample includes 3265 households in 2008 and 3352 households in 2014; it covers 409 Enumeration Areas (EAs) in 2008 and 419 EAs in 2014, with approximately 8 households interviewed in each EA. For just one exercise, we also draw on the nationally representative LSMS-ISA data collected in Nigeria in 2015/16. As in Tanzania, the Nigeria LSMS-ISA is designed to be representative of the urban and rural population at the national and zonal levels (Nigeria NBS 2015). Hereafter, the survey waves will be referred to by the year in which data collection began.

The Tanzania LSMS-ISA captures a rich set of information on household income-generating activities, indicators of welfare, and agricultural production. The definitions of some key variables used in this analysis are provided in Table A1. Of note, the calculation of crop and livestock income is inclusive of all agricultural production, including the imputed value of crops and livestock products that are consumed by the household.^3^ The share of farm income assessed in Table 7 includes income from crop and livestock production, as well as agricultural wage income. The survey includes a detailed consumption module that captures food consumption within the previous 7 days, as well as consumption of common nonfood products. The national poverty line is used with reference to the value of annualized consumption (not income) in order to categorize households as poor or nonpoor. The survey data include population weights, which are used in all analyses. In addition, real (inflation-adjusted) 2015 shillings are reported in all analyses.

The surveys report the official rural/urban classification of each household based on the national definition (to be detailed in Section 4). The households’ geographic coordinates are also captured using Global Positioning Systems (GPS). However, these coordinates are not reported precisely. Instead, the survey data include the average of the GPS coordinates for households sampled within each surveyed EA. (In Tanzania, urban EAs contain approximately 300–500 individuals (about 75–125 households), while rural EAs generally contain 700-900 individuals (about 135–175 households) and roughly follow village boundaries (Muzzini and Lindeboom, 2008; Wenban-Smith, 2015).) In Tanzania, approximately eight households were sampled within each surveyed EA in 2008 and 2014, while in Nigeria, approximately 20 households were surveyed within each EA. These numbers changed over time as migrants were tracked to new locations and some households dropped out of the sample. In addition, these EA coordinates are randomly offset by a value between 0 and 2 km in urban areas and 0–5 km in rural areas. For 1% of rural EAs, the offset value extends up to 10 km (Tanzania NBS 2012 and 2016; Nigeria NBS, 2015).

To construct alternative indicators of rural/urban status, we also draw from secondary data sources. These include the WorldPop data set (WorldPop, 2013) for information on local population density; the NOAA DMSP-OLS Nighttime Lights Time Series data set (NOAA, 2016) for information on nighttime light intensity; the Global Man-made Impervious Surface (GMIS) data set (Brown de Colstoun et al., 2017) for information on impervious surface cover; the Africapolis data set (2018) for spatial data on urban settlements; and views from Google Earth (2019).

## 4 Method

In this study, we explore whether the use of different definitions of “urban” and “rural” makes a meaningful difference in the measure of commonly referenced statistics (such as the level of urbanization) and in our understanding of whether rural Tanzania is undergoing transformation. Toward this end, we compare the level and pace of urbanization in Tanzania from 2008 to 2014 using 7 different rules-of-thumb to delineate the urban population. Because the relative levels of urbanization across countries is another common statistic cited in the economic development literature, we also apply our alternative definitions to compare this value for Tanzania and another country, Nigeria. We then return to Tanzania and limit our attention to three diverging urban definitions to understand whether they convey distinct stories regarding transformation in the rural population. Specifically, we evaluate the level and pace of change in rural welfare (with respect to the poverty level, among other indicators) and the reliance on agricultural income sources among rural households. We next narrow our attention further to the rural farming population and apply the three urban definitions to indicators of agricultural transformation (such as the level of farm commercialization). In a final exercise, we consider a different sort of decision around the urban definition to evaluate whether the manner in which places in Tanzania are officially recategorized as rural or urban over time makes a difference for the pace at which rural welfare is found to improve between 2008 and 2014.

The seven definitions or rules-of-thumb that are used to categorize households as being either rural or urban are outlined in Table 1. The first is the official administrative categorization.^4^ In Tanzania, the National Bureau of Statistics applies the concept to the smallest unit of analysis in the census, the EA. Regional and district headquarters are always considered to be urban, while the urban status of any EA beyond these administrative boundaries is determined subjectively by local census committees. Urban EAs usually have their own markets, schools, and health centers, while these features are commonly lacking in rural EAs. There are no strict criteria related to settlement size or population density in Tanzania, and the census committee essentially decides whether the EA “feels” urban (Muzzini and Lindeboom, 2008, p. 5). It is worth noting that EAs can be recategorized during the time of the census; this occurred in Tanzania in 2012, and the implications of this periodic recategorization will also be discussed in Section 5. Because we refer to the level of urbanization in Nigeria for one cross-country exercise, we also note the official urban definition in Nigeria, where settlements with populations of at least 20,000, along with all state capitals, are considered to be “urban” (Fox et al., 2018; Potts, 2017). Table 1 also notes the strengths and drawbacks of each urban definition used in this analysis. As noted in Section 2, while the administrative categorization may be contextually specific, the diversity of definitions limits the potential for cross-country analyses of urbanization or statistics related to urban or rural populations. The official classification of places as urban may also be influenced by political considerations or the allocation of fiscal resources (Bloch et al., 2015).

**Table 1:** Definitions of “urban” and “rural”.

		Definition/construction	Strengths	Limitations
1.	Administrative definition	The official designation in each country	Can be contextually specific (for example, accounting for high population densities in areas that are considered to be rural in nature)	Official definitions vary across countries (limiting the potential for cross-country comparisons) and sometimes are not clearly defined. Official categorizations may be influenced by political or other considerations.
2.	Population density	A household is categorized as urban if the local population density is at least 500 persons/km^2^ (from WorldPop).	Analysts can set their own threshold; can be consistent across countries	As a single indicator, this does not capture multidimensional understandings of “urban” spaces. Census data may be outdated.
3.	Impervious surface	A household is categorized as urban if the share of impervious surface cover is at least 2% (from the GMIS data set of Landsat).	Analysts can set their own threshold; can be consistent across countries	Small towns in low-income countries may have an urban character without the presence of impervious surfaces. This data set is available for only one year (2010).
4.	Night light intensity	A household is categorized as urban if the intensity of night light is at least 8 on a scale of 0–63 (from the NOAA DMSP-OLS Nighttime Lights Time Series data set).	Analysts can set their own threshold; can be consistent across countries	Small towns in low-income countries may have an urban character even without the energy infrastructure to support night lights. As of the time of writing, the data are not available in a readily useable format beyond 2013.
5	Africapolis	The designation of urban areas is provided by Africapolis, which bases its determination on the settlement population size (≥10,000) and the distance between buildings (within 200 m).	A consistent definition is applied in all countries, using information from censuses and satellite imagery. The outline of urban areas is made available in an easy-to-use format.	The rules used to identify urban spaces cannot be adjusted by the analyst. This data set is available for one year (2015).
6.	Local nonfarm economy	A household is categorized as urban if the average share of nonfarm income (excluding crop, livestock, or agricultural wage income) among the nearest 7 neighbors is at least 66%.	Explicitly measures the economic shift away from agriculture that commonly underpins the definition of “urban” spaces.	Requires detailed information on income-generating activities typically collected in resource-intensive (and infrequent) household surveys.
7.	Subjective assessment	A household is categorized as urban based on subjective assessment of Google Earth images. This labor-intensive categorization was applied only to the 2014 survey wave.	Can be contextually specific and multidimensional.	This method is labor-intensive and, by definition, subjective. Research assistants found it difficult to estimate with confidence the presence of amenities in the aerial view of communities.

The second rule used to categorize households as rural or urban is based on local population densities, applying a threshold of 500 persons/km^2^ to identify urban areas (following the example of Siri et al., 2008). Population density values were extracted for the GPS coordinates and for each survey year from WorldPop (2013).^5^ The “local” measure is arrived at by using the finest resolution geographic data available (in the case of WorldPop, this is approximately 100 m^2^ at the equator) and measuring the mean value of population densities found within either a 2.5 km radius (in officially rural areas) or a 1 km radius (in officially urban areas). This is intended partly to accommodate the random offsets of GPS coordinates, as described in Section 3.

An urban definition based on population density has the advantage of having a flexible threshold, and analysts can conduct sensitivity analyses with the threshold selected. In Section 5, we discuss the implications of selecting a different urban threshold for continuous variables. Our intent is not to argue that one threshold is more appropriate than others, but rather to explore whether the use of reasonable thresholds across different urban definitions may lead to different conclusions. WorldPop necessarily relies on the most recent census data to produce its population density estimates. However, these may be quite outdated and, while WorldPop population densities seem to be adjusted over time to account for country-level population growth, the estimated rate of population growth does not appear to vary over space. As noted in Section 2, another drawback of an urban definition based only on population density is that densely populated rural communities with agricultural economies may be categorized as urban, based on this single metric.

**Fig. A1:**
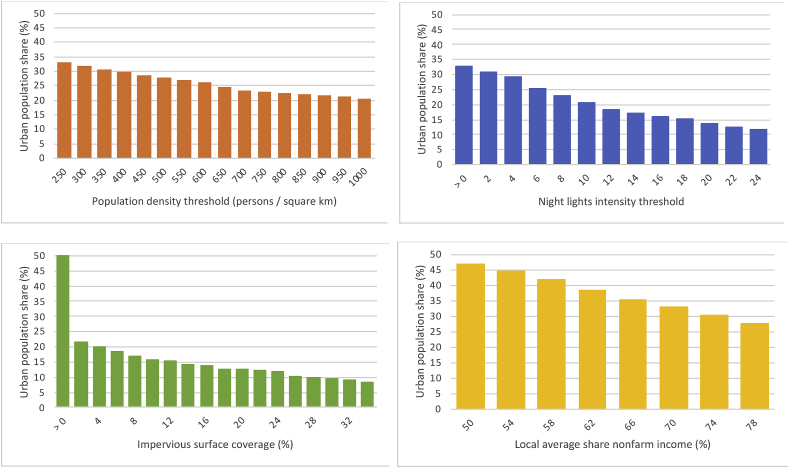
Sensitivity analysis for thresholds used in urban designations (Tanzania)

The third urban definition used in this analysis is based on local levels of impervious surface cover, which refers to surfaces through which rainfall cannot pass. We apply a threshold of 2% impervious surface cover to identify urban areas, with these values extracted from the Global Man-made Impervious Surface (GMIS) Dataset from Landsat (Brown de Colstoun et al., 2017). Though this threshold seems low, it was selected considering the pattern seen in Figure A1, where there seems to be a sharp drop-off in the size of the detected urban population at a value of 2%. This data set is constructed from daytime satellite imagery from 2015 and is linked to all survey waves. Analysts can set their own thresholds when using this definition to delineate urban spaces. However, it seems possible that emerging urban centers in low-income countries may lack impervious surfaces, casting doubt on the appropriateness of this definition in poor countries. As will be discussed in Section 5, this definition tends to produce conservative estimates of the rate of urbanization in both Tanzania and Nigeria. Another drawback of this data set is that it is only available for one year (2015), and while it may provide an approximate measure of impervious surface cover for a given location in the years before and after 2015, it cannot be used to track the emergence or growth of urban spaces over time.

The fourth urban definition used in this analysis is based on the intensity of nighttime light, as captured by satellite imagery. Night light values are extracted from the NOAA DMSP-OLS Nighttime Lights Time Series data set (NOAA, 2016). The intensity of night light is reported in “digital numbers” on a scale from 0 to 63, and we apply a threshold of 8 to categorize brightly-lit places as urban and unlit places as rural, reflecting the slight inflection point observed in Figure A1.^6^ To our knowledge, these annual data sets are available only up to 2013, and the waves of survey data are linked to the closest available year of night light data. Analysts can set their own thresholds when using an urban definition based on night lights. However, as with impervious surface cover, emerging urban centers in low-income countries may plausibly have an urban “feel” yet still lack an electrical system. Relying on this metric alone would undercount these less established urban spaces.

The fifth criteria used in this analysis comes from Africapolis (2018), which has produced a standardized spatial data set of urban centers across Africa, categorizing places as urban with consideration of settlement population size (≥10,000) and distance between buildings (within 200 m). We consider a household to be urban if it falls within an urban polygon, as drawn by Africapolis. Their analysis is based on the most recent census data available in a given country and reflects the year 2015. This geographic data set is relatively easy to use in conjunction with geographically explicit household survey data. However, as noted earlier, the most recent census data in a given country may be outdated, and it is not entirely clear how population numbers reported at different levels (wards in Tanzania or Local Government Areas in Nigeria) are linked to newly drawn urban shapes. Furthermore, analysts cannot make their own decisions and adjust the thresholds used to delineate urban spaces.

The sixth rule used in this analysis is based on the extent to which the local economy is oriented towards agriculture, and this information is drawn entirely from within the LSMS-ISA household survey. A household is categorized as urban if the average share of nonfarm income (excluding crop, livestock, or agricultural wage income) among its nearest neighbors in the data set is at least 66%. In Tanzania, we refer to the 7 nearest neighbors, as this generally reflects the other households sampled in an EA. In Nigeria, we refer to the 19 nearest neighbors for the same reason. This definition produces a fairly stable categorization of EAs over the years when considering the average share of nonfarm income among all resident households. Specifically, in Tanzania 87% of EAs remain either urban or rural over three panel survey waves (2008, 2010, and 2012), while this value is 76% in Nigeria. A strength of this urban definition is that, as noted in Section 2, a shift away from agriculture is an important aspect of urbanization, and this is the only definition to capture this explicitly. However, this definition necessarily draws from resource-intensive household surveys that may not be available in all settings and years. Furthermore, it is not obvious what is the threshold for average nonfarm income shares that renders a community “urban”. As will be discussed in Section 5, using a threshold of 66% tends to produce relatively more liberal measures of urbanization in Tanzania and Nigeria.

**Fig. A2:**
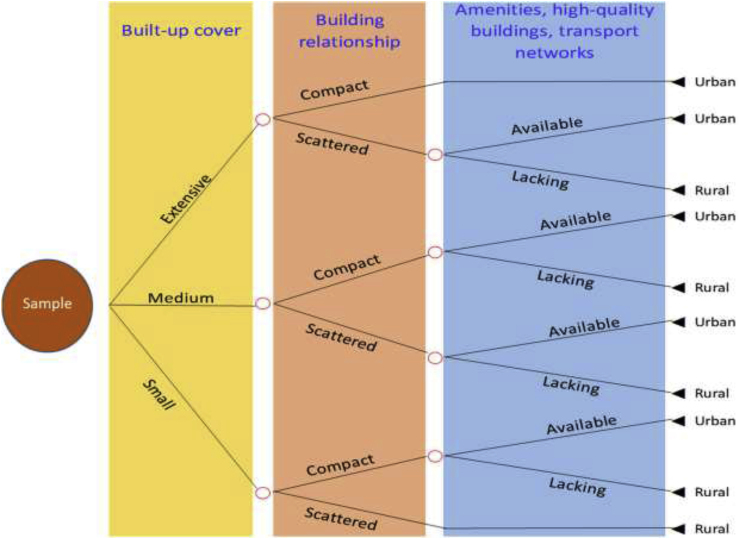
Galdo et al. (2018) decision tree for subjective assessments of Google Earth images Source Copied directly from Galdo et al. (2018)

**Fig. A3:**
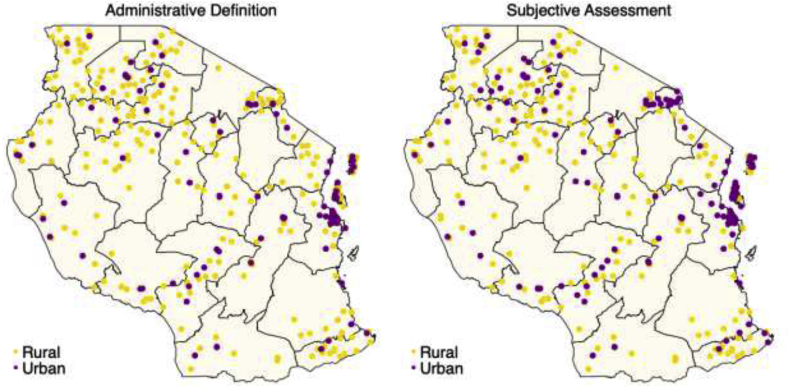
Categorization of 2014 enumeration areas with two urban definitions

The seventh criterion used to categorize households as urban or rural is based on subjective judgment. For the 2014 survey wave in Tanzania, we follow the example of Galdo et al. (2018) and visually inspect images from Google Earth. For visual clarity, we hover over the GPS coordinates and zoom to an estimated scope of vision of 2 km across. We then assess whether the sites seem to have extensive built-up cover, compact buildings, and visible roads. This assessment was conducted by three research assistants, and the decision tree of Galdo et al. (2018) was applied to categorize each image as urban or rural (see Figure A2). For 87% of the sites, all researchers arrived at a consistent urban or rural categorization. In cases where there was internal disagreement with regard to the final categorization, we applied the urban/rural category selected by the majority. An illustration of the categorization of enumeration areas in 2014 according to the administrative and subjective criteria is presented in Figure A3. The strength of this urban definition is that it captures multiple dimensions of urban spaces and can be applied in a fairly simple fashion (with access to Google Earth). At the same time, applying this definition is rather labor-intensive, and researchers may find it difficult to relate an aerial view of a place to what the community likely feels like on the ground.

## 5 Results

The various urban definitions delineated in Section 4 are now assessed within some standard analyses of household survey data. To address research question 1 (‘How do different urban definitions affect the estimated level and pace of urbanization in Tanzania?‘), Table 2 presents the estimated share of the population residing in urban areas for the most recent survey year, using each urban definition in turn. In Tanzania, the official urban/rural categorization found in the LSMS-ISA data set leads us to conclude that 28% of the national population is urban. This is quite consistent with the urban population share as measured with a population density-based definition (at 29%), and it is also consistent with the Government of Tanzania's official estimate of the level of urbanization around this time (at 30% in 2011, as estimated with the 2012 Population and Housing Census (Tanzania National Bureau of Statistics NBS, 2013)). However, this value differs when we apply different urban definitions.^7^ An impervious surface-based definition paints a picture of a country that is considerably less urban at 21% (a difference of an estimated 3.71 million residents). Similarly, with a night light definition, 23% of the population is categorized as urban. Applying the geographic outlines of urban areas from Africapolis to this data set produces an urban population share of 27%.^8^ Meanwhile, focusing on the nonfarm orientation of the local economy reveals a country that looks far *more* urbanized at 35% (a difference of an estimated 3.22 million residents).

**Table 2:** Levels and rates of urbanization in Tanzania (%).

	Urban population share, 2014	Δ urban population share, 2008 to 2014
Administrative definition	28	6
Population density	29	8
Impervious surface	21	5
Night light	23	11
Africapolis	27	7
Local nonfarm economy	35	11
Subjective assessment	39	N/A

We also consider whether the estimated pace of urbanization varies across these urban definitions. According to the administrative definition, 6% more of the Tanzania population resided in urban areas in 2014, as compared with 2008. However, this rate is lower with an impervious surface cover definition (at 5%) and higher when focusing on the nonfarm orientation of the local economy (at 11%).

In Table 3, we consider the alignment of rural and urban household categorizations in Tanzania across the administrative (official) definition and the other definitions. The population density-based categorization produces categories that are consistent with the administrative categorization for 90% of households. At the same time, 5% of households are considered to be urban by the local definition, though their local population density is less then 500 persons/km^2^. Nearly one-fifth (19%) of households are officially considered to be urban but reside in places with an impervious surface cover of less than 2%. Across most definitions, it is uncommon for households to be recategorized from rural to urban, indicating that the urban definition currently applied in Tanzania is fairly liberal. This does not support the argument that there is considerable under-recognition of urban spaces (Muzzini and Lindeboom, 2008). The only exceptions are when using a local economy criterion (with 9% of households recategorized from rural to urban) and when relying on subjective assessment (with 13% of households recategorized from rural to urban).

**Table 3:** Cross-tabulation of rural and urban categories (%).

		Administrative definition (2014)	
		Rural	Urban
**Population density**	**Rural**	62	5
	**Urban**	5	28
**Impervious surface**	**Rural**	66	19
	**Urban**	0	15
**Night light**	**Rural**	66	10
	**Urban**	1	24
**Africapolis**	**Rural**	62	7
	**Urban**	5	26
**Local nonfarm economy**	**Rural**	57	2
	**Urban**	9	32
**Subjective assessment**	**Rural**	54	2
	**Urban**	13	32

**Fig. 1:**
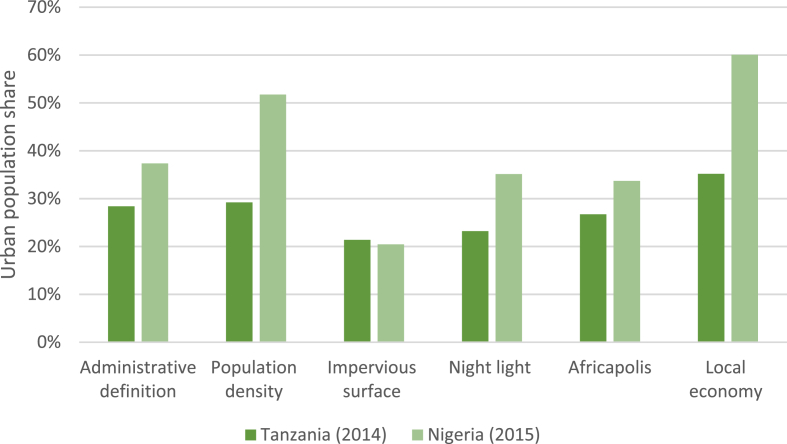
Cross-country comparison of urbanization levels in Tanzania and Nigeria.

Policy makers, such as donors, may sometimes make cross-country comparisons when deciding where to allocate resources. For this reason, we now explore research question 2, ‘How do different urban definitions affect comparisons of urbanization across two countries, Tanzania and Nigeria?’ When comparing the level of urbanization in Tanzania to that of another country, Nigeria, we find that the ordering of urbanization levels sometimes does reverse with some urban definitions (Fig. 1). Thus, using the administrative definitions in each country, Nigeria is considered to be more urbanized than Tanzania at 37%.^9^ However, when applying a definition based on impervious surface cover, a slightly *smaller* share of the population in Nigeria (at 20%) is considered to reside in an urban area. When using the Africapolis definition, Nigeria appears to be more urbanized than Tanzania by 7 percentage points, but when using the local economy definition, this value grows to 25 percentage points.^10^ This finding suggests that donors and policy makers ought to be cautious when comparing rural or urban statistics across countries.

Turning to research question 3 (‘How does the application of different urban definitions in Tanzania affect measures of welfare and economic orientation in the rural population?‘), we next consider indicators of welfare in rural Tanzania, such as consumption levels and education outcomes. As noted in Section 2.2, rural welfare is expected to improve in the process of rural transformation. For this exercise (and with consideration of space constraints), we focus on a subset of three definitions that seem most distinct from one another. Results, reported in Table 4, show that some variables are consistent across definitions. For example, in Tanzania, the measured poverty rate among rural households is 30% with the night light definition and 34% with the local economy definition, and these are not statistically significantly different from the 32% measured with the official definition. At the same time, the estimated share of rural households with electricity varies dramatically from 3% (with the local economy definition) to 9% (with the night light definition).

**Table 4:** Indicators of welfare in the rural population.

	Mean values, 2014			Δ in mean, 2008-14		
	Administrative	Night light	Local nonfarm economy	Administrative	Night light	Local nonfarm economy
Value of consumption (1,000s TSh/AE/day)^a^	2.60	2.78**	2.48*	0.15**	0.11	−0.01
1 = Poor	0.32	0.30	0.34	0.00	0.01	0.04**
Proportion of food purchased	0.58	0.61***	0.55**	0.10***	0.07***	0.07***
Proportion of budget spent on food	0.76	0.75*	0.77	−0.02***	−0.01***	−0.01*
1 = Any household member completed primary school	0.74	0.74	0.72	0.05***	0.02	0.02
1 = Modern roof materials	0.65	0.67	0.62*	0.17***	0.14***	0.13***
Time to access water in dry season (minutes)	50.67	49.40	53.03	−17.49***	−15.90***	−15.84***
1 = Electricity	0.07	0.09**	0.03***	0.05***	0.04***	0.01*
Obs.	1984	2216	1787			

Note: Asterisks in columns 2 and 3 denote the level of statistical significance for a *t*-test of equality of mean values in this column and column 1. Asterisks in columns 4, 5, and 6 denote the level of statistical significance for a *t*-test of equality of mean values in the first and most recent survey wave, using the rural definition in a given column. ***p < 0.01, **p < 0.05, *p < 0.1.

a These are real (inflation-adjusted) 2015 shillings.

Across these different definitions, we sometimes do find diverging trends over time from 2008 to 2014. When using the administrative definition, the average value of consumption in rural households increased by an estimated 150 shillings/adult equivalent/day (a 6% increase from the average level in 2008). However, this value actually decreased over time (though not statistically significantly) when using the local economy definition. Similarly, although we usually find a positive trend over time in the rate at which any household member has completed primary school, this trend is not statistically significant when using the local economy definition. Recall that with this definition, an initially rural household is reclassified as urban as soon as its neighbors draw at least 66% of their income, on average, from off-farm sources. Assuming that off-farm activities are correlated with higher incomes, this means that rural areas that become wealthier are often reclassified as urban, leaving only those areas that remain economically stagnant as “rural”. Thus, it is not surprising to find that rural welfare is not found to improve when the rural population is delineated using a definition based on the local economy.

In Table 5, we unpack these differences by focusing on two definitions (the administrative and night light definitions) and comparing the welfare characteristics of households that are categorized as rural under both definitions to those that shifted between rural and urban categories. Compared to households that remained rural (column 1), those that had been considered rural under the local definition but are considered urban with the night light definition, and those that had been considered urban but are recategorized as rural with the night light definition, are statistically significantly wealthier. These shifters have higher values of consumption and lower rates of poverty; they spend less of their budget on food and access more of their food through purchases; and they are more likely to live in homes with modern roof materials. However, note that households that are recategorized as rural (column 3) are wealthier (and greater in number) than those that are recategorized as urban (column 2). This explains why, in Table 4, we observed that the night light-based rural population seemed wealthier than the rural population as defined with the administrative (official) definition.

**Table 5:** Welfare indicators of households that shift rural/urban categories between administrative and night light designations (mean values, 2014).

	Rural (administrative) - rural (night light)	Rural (administrative) – urban (night light)^a^	Urban (administrative) - rural (night light)^a^
Value of consumption (1,000s TSh/AE/day)	2.57	3.98***	4.44***
1 = Poor	0.32	0.05***	0.16***
Proportion of food purchased	0.57	0.86***	0.88***
Proportion of budget spent on food	0.77	0.71***	0.67***
1 = Any member completed primary school	0.73	0.96***	0.78
1 = Modern roof materials	0.64	0.98***	0.85***
Time to access/fetch water in dry season (minutes)	51.64	3.43***	31.41***
1 = Electricity	0.05	0.74***	0.38***
Obs.	1912	72	304

a Asterisks denote the statistical significance of a two-sample *t*-test for equality of means between column 1 (rural-rural) and column 2 or 3. ***p < 0.01, **p < 0.05, *p < 0.1.

As discussed in Section 2.2, with structural transformation we would expect to see a shift away from a reliance on farm income, even within the rural population (Barrett et al. 2017a, 2017b). We next explore the income portfolios of rural households in Table 6, especially focusing on the income shares derived from crop production, livestock production, and agricultural wages, which together comprise the household's share of farm income.^11^ In Tanzania, across the three definitions analyzed here, we find that the average household income share from crops varies from 37% with a night light definition to 42% using the local economy definition. The average share of income from all agricultural sources is 60% using the official (administrative) definition, 57% with the night light definition, and 64% using the local economy definition. With the administrative and night light definitions, it is evident that rural households are increasingly shifting away from agriculture. However, this trend is not significant specifically with the local economy definition. As less agriculturally-focused areas are recategorized as urban with this definition, it makes sense that those who remain “rural” appear to be static—These are, by definition, the areas that are unchanging.

**Table 6:** Income shares in rural population (proportions).

	Mean values, 2014			Δ in mean, 2008-14		
	Administrative	Night light	Local nonfarm economy	Administrative	Night light	Local nonfarm economy
Crop	0.40	0.37**	0.42	−0.05***	−0.04***	−0.05***
Livestock	0.11	0.10	0.12	−0.02***	−0.01***	−0.01***
Ag wage income	0.10	0.10	0.10	0.04***	0.04***	0.05***
Self-employment	0.19	0.21	0.17*	−0.01	−0.02**	−0.02**
Non-ag wage income	0.11	0.12	0.09*	0.03***	0.01***	0.01**
Transfers	0.08	0.09	0.09	0.03***	0.03***	0.03***
Other	0.01	0.01	0.01	−0.02***	−0.02***	−0.01***
Share farm income	0.60	0.57**	0.64**	−0.02***	−0.01*	−0.01
Obs.	1968	2203	1776			

Note: Asterisks in columns 2 and 3 denote the level of statistical significance for a *t*-test of equality of mean values in this column and column 1. Asterisks in columns 4, 5, and 6 denote the level of statistical significance for a *t*-test of equality of mean values in the first and most recent survey wave, using the rural definition in a given column. ***p < 0.01, **p < 0.05, *p < 0.1.

Our fourth research question is, ‘How does the application of different urban definitions affect measures of agricultural indicators within the rural farming population?’ As noted in Section 2.2, agricultural transformation is characterized by the adoption of modern farm inputs and practices, improved agricultural productivity, and a more commercial orientation of farms (Timmer, 1988). Table 7 presents some characteristics of farm-households in rural Tanzania, using alternative delineations of the rural population. Across the three definitions explored here, the share of rural households that engage in farming ranges from 88% (with the night light definition) to 94% (with the local economy definition). It follows that the extent to which agriculture is predominantly a rural practice also varies across urban definitions. However, when we focus on the population of rural farming households, we find no statistically significant differences in indicators of agricultural progress across these definitions. For example, the average rural farm size is 2.46 ha (as measured using the night light definition) or 2.53 ha (with the local economic orientation definition), and these are not statistically significantly different from the average rural farm size (2.52 ha) when using the administrative definition. From 2008 to 2014, farmers were 8–9% more likely to purchase improved seed and 2–4% more likely to use a tractor for land preparation. While average land productivity is unchanged over this period, labor productivity is seen to rise in a statistically significant manner across all three urban definitions. As expected during a process of agricultural transformation, farmers also seem to be selling a greater share of their agricultural production, with the average share increasing by 5% or 6% from 2008 to 2014 when applying all urban definitions. And among crop sellers, the share that sold some crops at the farm gate increased by 9% or 11% across all definitions. This pattern suggests that definitional decisions especially affect the categorization of non-farming (though possibly rural) households but are less consequential for the rural farming population.

**Table 7:** Characteristics of farms in rural areas.

	Mean values, 2014			Δ in mean, 2008-14		
	Administrative	Night light	Local nonfarm economy	Administrative	Night light	Local nonfarm economy
1 = Agricultural household	0.91	0.88***	0.94***	−0.07***	−0.05***	−0.03***
***Among agricultural households:***						
Land size (ha)	2.52	2.46	2.53	0.06	0.08	0.22
1 = Purchases improved seed	0.25	0.25	0.24	0.09***	0.08***	0.08***
1 = Uses tractor for land preparation	0.07	0.07	0.05	0.04***	0.04***	0.02***
Land productivity (value crop production/ha cultivated, main season - millions TSh)^a^	0.38	0.38	0.38	0.00	0.00	0.00
Labor productivity (value crop production/labor day, main season - TSh)^b^	4619.31	4599.94	4574.15	718.24***	725.68***	582.96***
Distance to agricultural market (km)	10.13	9.87	10.21	−0.30	−0.44	−0.27
Proportion agricultural production sold	0.43	0.42	0.43	0.06***	0.06***	0.05***
1 = Sells crops at farm gate	0.67	0.68	0.67	0.11***	0.10***	0.09***
Obs. (agricultural households)	1763	1911	1663			

Note: Asterisks in columns 2, 3, and 4 denote the level of statistical significance for a *t*-test of equality of mean values in this column and column 1. Asterisks in columns 5, 6, 7, and 8 denote the level of statistical significance for a *t*-test of equality of mean values in the first and most recent survey wave, using the rural definition in a given column. ***p < 0.01, **p < 0.05, *p < 0.1.

a Area-adjusted weights are applied.

b Labor day-adjusted weights are applied.

In a final exercise, we tackle our fifth research question, ‘How does the recategorization of places as rural or urban over time affect the rate at which rural welfare is found to improve or deteriorate in Tanzania?’ As discussed in Section 4, enumeration areas (EAs) in Tanzania are re-assessed with each census to determine their categorization as rural or urban. A local census committee judges whether each EA includes a market, school, and/or health center in the community, and essentially whether it “feels” urban (Muzzini and Lindeboom, 2008, p. 5). The most recent censuses in Tanzania were in 2002 and 2012 (the latter falling within the time frame of the LSMS-ISA data set). What does this recategorization mean for our understanding of trends in rural welfare? If the most successful rural areas are consistently recategorized as urban, and if measures of rural welfare are captured with regard to the rural/urban categorizations in a given year, rural poverty would appear to plateau or decline more slowly than if the rural/urban categorizations over space were frozen at the baseline year. In other words, it is *by definition* that measures of rural welfare would remain more or less unchanged (Goetz et al., 2018; Van Dam, 2019).

We attempt to explore this by referring to the rural/urban status of EAs in the 2014 survey wave (after EAs were recategorized in the 2012 census) and comparing this to the status of wards in 2002 (prior to the recategorization). A ward is an administrative level just above village/enumeration area, and wards therefore contain multiple EAs. Note that ward boundaries are redrawn (and the number of wards proliferate) with each census. Ideally, we would want to know the rural/urban categorization of each specific location before 2012. However, we were only able to access data at the ward level, with wards categorized as entirely rural, entirely urban, or mixed (containing both urban and rural EAs). Fifteen percent of wards were categorized as mixed in 2002; thus, our analysis is necessarily imprecise. We assume that EAs that were rural in 2014 were also rural in 2002. Because we cannot speculate on the prior rural/urban status of EAs that were urban in 2014 but fall within a ward that had been mixed in 2002, we conservatively focus only on the 136 households (2.5%) that were urban in 2014 but fall within a ward that was entirely rural as of 2002.

Table 8 reports the average change in mean values of welfare indicators in rural Tanzania. The first column uses the local (administrative) urban definition in 2008 and 2014. (These results were reported previously in Table 4.) In the second column, the rural population in 2014 is considered to be inclusive of those households categorized as rural in 2014, along with currently urban households that would have been categorized as rural in 2002. In other words, to the best of our ability, we attempt to hold the geographic boundaries of rural Tanzania constant in both 2008 and 2014. The results show that, if we did not allow rural Tanzania to physically shrink over time, it would seem that poverty declined slightly faster (by one percentage point), the rate of primary school completion increased faster (by one percentage point), and access to electricity increased faster (by two percentage points). We stress that this is a conservative assessment of the impact of the periodic EA recategorization on trends in rural poverty. Nevertheless, by measuring rural welfare in 2014 using the baseline rural/urban status of EAs, we arrive at a slightly more positive story of rural poverty reduction.

**Table 8:** Trends in rural welfare with time-variant and time-constant rural/urban categorizations over space.

	Δ in mean, 2008–14 (Administrative definition)	
	Time-variant categories based on the 2002 and 2012 censuses	Time-constant categories based on the 2002 census
Value of consumption (1,000s TSh/AE/day)	0.15	0.23
1 = Poor	0.00	−0.01
Proportion of food consumed that was purchased	0.09	0.11
Proportion of budget spent on food	−0.02	−0.02
1 = Any household member completed primary school	0.04	0.05
1 = Modern roof materials	0.17	0.18
Time to access/fetch water in dry season (minutes)	−17.49	−18.84
1 = Electricity	0.04	0.06

## 6 Summary and conclusions

This paper is motivated by the question of how our understanding of urbanization and processes of transformation is influenced by decisions around how we define “rural” and “urban”. We alternately apply seven rules or criteria to categorize households in Tanzania, drawing from a wide set of data sources and types, including daytime and nighttime satellite data and household survey data, and apply reasonable thresholds in each case to segment the population. These different categorizations are used in some common analyses of urbanization and economic/demographic change in rural populations. The focus on Tanzania is intended to be illustrative, and the lessons learned from this exploration of the Tanzanian context are assumed to be relevant in other low-income countries. Nevertheless, the patterns unearthed in this study may manifest somewhat differently in other settings.

With regard to research question 1, the measured level (and pace) of urbanization in Tanzania varies with different urban definitions, and this could plausibly affect relative budget allocations for government ministries or programs that are oriented specifically toward urban or rural populations. Across these definitions, it is usually more common for officially urban households in Tanzania to be reclassified as rural (as opposed to the reverse). With regard to research question 2, the divergence in urbanization levels between Tanzania and another country (Nigeria) is quite sensitive to which urban definition is applied. This could have implications for international development organizations if they sometimes base their investment decisions on cross-country comparisons of urbanization and the characteristics of rural or urban populations. Note that different countries are likely to apply different urban definitions in their official statistics.

With regard to research question 3, we find that the urban definition sometimes (though not always) affects some common analyses of welfare and economic orientation in the rural population. For example, the share of the rural population with electricity is estimated to be 3% with a local economy-based definition but 9% with the definition based on night light. This is because the night light definition recategorizes relatively wealthy households from urban (with the administrative definition) to rural (with the night light definition). Nevertheless, when it comes to research question 4, we find that these different definitions paint a fairly consistent picture of the population of rural farm-households in terms of levels of engagement with input and output markets. It therefore seems that the urban definition is not so consequential for studies of the rural farming population, which is often the focus of analyses of agricultural transformation.

This paper further highlights the implications of the periodic recategorization of places as rural or urban with each census (research question 5). The Food and Agriculture Organization of the United Nations emphasizes their goal to reduce rural poverty worldwide and is therefore likely to be interested in tracking progress toward this goal (FAO, 2019). However, we show that the detected rate of progress is sensitive to whether we consider rural Tanzania to be fixed in space or to change shape over time. In fact, rural poverty would appear to decline faster—a more optimistic narrative—when holding the rural boundaries constant.

We hope this paper will inspire additional thought regarding how “urban” *ought* to be defined in a given context, and also whether and when it is appropriate to define “urban” consistently across different contexts. López Moreno (2017), for example, suggests applying a universally standardized definition and measurement of the ‘urban extent’ when monitoring progress toward achieving Sustainable Development Goal 11 to “make cities and human settlements inclusive, safe, resilient and sustainable.” If using the local definition in a given country, analysts should be aware of how rural/urban categories have been generated and should consider whether their conclusions may differ with a different data source or if a different categorization rule were applied. Along the same lines, policy makers should give attention to the definitions that underpin any statistics used in their decision making.

Finally, analysts may not realize the wealth of data, including remotely sensed data, that are publicly available. A description of the data sources used in this paper is available in the appendix, and the code used in this analysis is also available for dissemination to help other analysts make thoughtful decisions around the urban definitions in their own research.

## Funding

This work was supported by The Bill and Melinda Gates Foundation [OPP1135685].

## CRediT authorship contribution statement

**Ayala Wineman:** Project administration, Conceptualization, Methodology, Formal analysis, Writing - original draft, Visualization. **Didier Yélognissè Alia:** Conceptualization, Methodology, Data curation, Software, Writing - review & editing. **C. Leigh Anderson:** Supervision, Funding acquisition, Conceptualization, Methodology, Writing - review & editing.

## Declaration of competing interest

None.

## Figures and Tables

**Table A1:** Definitions of variables used in analysis

Table or Figure	Variable	Definition/Construction
Table 4, Table 5	Value of consumption (1,000s TSh/AE/day)	Household consumption is the sum of the annualized monetary value of food products consumed within the past week, the annualized amount spent on other commonly purchased products within the previous month, and the amount spent on less commonly purchased goods over the past year. This is inclusive of both market and nonmarket transactions. These values are weighted with a Fisher food price index specific to geographic stratum and quarter to reflect the cost of living across different settings in Tanzania. The household consumption aggregate was produced by the World Bank and made available with the data set. These values are divided by the number of adult equivalents (AEs) in the household and by the number of days in a year. These are reported in real (inflation-adjusted) 2015 Tanzanian shillings (TSh).
	1 = Poor	Household poverty status is defined relative to the national poverty line in Tanzania (World Bank, 2015). A household is categorized as poor if the value of household consumption per adult equivalent per day falls below 1588 shillings in real (inflation-adjusted) 2015 TSh. With a 2015 exchange rate of approximately $1 USD = 2207 TSh, this equals $0.72.
	Proportion of food purchased	The share of the value of food consumed by the household over the previous 7 days that was acquired through purchase
	Proportion of budget spent on food	The share of a household's annual value of consumption (of food and nonfood items and expenses) that is allocated towards food
	1 = Any member completed primary school	Any household member has completed primary school (Standard 7)
	1 = Modern roof materials	The roof materials of the household dwelling is made of metal sheets, concrete/cement, tiles, or other modern materials
	Time to access water in dry season (minutes)	Respondent-estimated number of minutes (round trip) to access water in the dry season
	1 = Electricity	The household's source of energy for lighting is electricity
Table 6	Income shares:	
	Crop	Crop income includes the value of crop sales, as well as the imputed value of crops produced and consumed. Crops retained for own consumption are valued either at the observed sales per kg (if the household sold any of a given crop), or at the median per-kg sales price at the smallest geographic unit for which at least 10 sales are observed in the data set in a given year. This is net of the value of costs associated with crop production.
	Livestock	Livestock income includes the value of livestock and livestock products sold, as well as the imputed value of livestock and livestock products that were produced and consumed. Values are imputed using the same method as for crop values above. This is net of the value of costs associated with livestock production.
	Agricultural wages	Income earned from working on others' farms
	Self-employment	Net income from businesses of all sizes (revenue minus business expenses)
	Non-agricultural wages	Income earned from working for others, whether in a salaried or wage position
	Transfers	Remittances, pensions, and any other financial assistance received
	Other	Any other sources of income, such as rental income
	Share farm income	Farm income is inclusive of income from crop and livestock production, as well as agricultural wage income.
Table 7	1 = Agricultural household	A household is considered to be agricultural if it has cultivated any farmland or holds any livestock.
	Land size (ha)	To compute the size of a household's landholdings, we use the Global Positioning Systems (GPS) area measures that are available for approximately three-quarters of cultivated plots, and the sizes of unmeasured plots are imputed with reference to farmers' estimates of land size and local patterns of measurement error.
	1 = Purchases improved seed	The farm-household cultivates improved seed that was newly purchased.
	1 = Uses tractor for land preparation	The farm-household prepares land for planting using a tractor.
	Land productivity (value crop production/ha cultivated)	The value of crop production in the main growing season (with crop values estimated as with crop income above), divided by area cultivated in the main season. This is reported in millions of real 2015 shillings, and the average value is calculated using area-adjusted weights (population weights multiplied by the area cultivated).
	Labor productivity (value crop production/labor day)	The value of crop production in the main growing season, divided by the number of labor days allocated to the farm in the main season. This is reported in real 2015 shillings, and the average value is calculated using population weights multiplied by number of labor days.
	Distance to agricultural market (km)	Farmer-reported distance to the nearest agricultural market. These were provided for each plot, and the value for the largest plot is used here.
	Proportion agricultural production sold	The share of the value of agricultural production (inclusive of crop and livestock production) that is marketed, as of the time of survey.
	1 = Sell crops at farm gate	Crops are sold at the farm, rather than transported elsewhere for sale.

## Appendix B

### Geographic Data Sources and Instructions for Extraction

#### POPULATION DENSITY

**Source:** WorldPop.

**Website:** http://www.worldpop.org.uk.

**Note:** Population density data can be downloaded for a given country at different spatial resolutions. In this paper, we use the finest resolution available, that being approximately 100 m at the equator. The values represent persons per grid, which is roughly the same as persons per hectare.

**Resolution:** 100 m (0.000833333 decimal degrees; 3 arc).

**Date of production:** 2018.

**Variable:** Persons per grid square in a given year.

#### GLOBAL MAN-MADE IMPERVIOUS SURFACE (GMIS)

**Source:** NASA Socioeconomic Data and Applications Center (SEDAC).

Website: http://sedac.ciesin.columbia.edu/data/set/ulandsat-gmis-v1.

**Note:** The GMIS data set is accompanied by the Global Human Built-up and Settlement Extent (HBASE) data set. GMIS data can be downloaded for a given country at different spatial resolutions. In this paper, we chose to use the finest resolution available, that being 30 m. The values represent the share of the grid that is covered with impervious surfaces.

**Resolution:** 30 m.

**Date of production:** 2017.

**Reference period:** The GMIS data set reflects conditions in 2010.

**Variable:** Percent of area covered by impervious surfaces (i.e., water cannot seep through).

#### NIGHT LIGHT INTENSITY

**Source:** NOAA Defense Meteorological Satellite Program (DMSP) — Nighttime Lights Time Series 1992–2013 (Version 4).

**Website:** https://ngdc.noaa.gov/eog/dmsp/downloadV4composites.html.

**Note:** This data set is global and cannot be downloaded separately for a given country. The data set seems to be available for 1992–2013, and we have not located night light data for more recent years. The values represent a measure of light intensity (range 0–63). These are stable lights.

**Resolution:** 30 arc second grids (1 km at the equator).

**Variable:** Light intensity (range 0–60).

#### AFRICAPOLIS

**Source:** Africapolis, produced by the OECD Sahel and West Africa Club in collaboration with e-geopolis.

**Website:** http://www.africapolis.org/home.

**Date of production:** 2018.

**Reference period:** The Africapolis data set contains estimates for 2015, using the most recent census data available in each country.

**Note:** This geospatial database is in the form of polygons representing urban centers. In this project, we identify whether geographic coordinates are found within an Africapolis urban polygon in Stata, using the following user-written commands: <shp2dta> (to convert the shapefile to a Stata dataset) and <geoinpoly> (to match the coordinates to urban polygons). Our Stata code is available to readers upon request.

#### Google Earth

**Website:** www.google.com/earth/

**Note:** We used R to interact with Google Earth and create a set of files (images) of consistent zoom for each site.

### ESTIMATING LOCAL AVERAGE VALUES OF GEOGRAPHIC DATA

The R script that we used to extract local average values of population density, GMIS, and night light intensity is available to readers. The R script generates the average value of a given variable (for example, population density) over all grids that are found within a specified radius of the latitude and longitude. In our script, we specify a radius of 1 km for sites that are officially considered to be urban and 2.5 km for sites that are officially considered to be rural. This decision is intended to reflect the random offsets of the geocoordinates in these two categories. The data files that are produced therefore include, for a given point, the average of a variable (like population density) within either 2 or 5 km.

### GATHERING GOOGLE EARTH IMAGES

The R script that we used to collect images from Google Earth is also available to readers. Our script specifies a zoom value of 15. We estimate that this is roughly equal to capturing an image of 2 km across. This value is selected for all sites (i.e., those that are officially considered to be both rural and urban) because a larger diameter interferes with visual clarity when classifying these images.

## Footnotes

1 Some authors argue that a binary urban-rural dichotomy is increasingly obsolete as improved transportation and communication systems blur the related distinctions (Lerner and Eakin, 2011; Cohen, 2004; Berdegué and Proctor, 2014). Nevertheless, governments, development agencies, and other policy makers continue to work with the labels of “rural” and “urban”. To engage with contemporary policy discussions, Potts (2017, 2018) argues that it remains necessary to consider and refine these terms.

2 This data set is publicly available and can be accessed through the World Bank: http://surveys.worldbank.org/lsms/programs/integrated-surveys-agriculture-ISA/tanzania.

3 As detailed in Table A1, the value of own consumption from agricultural production is imputed using observed sales prices, consistent with the approach used by the FAO Rural Income Generating Activities project (FAO n.d.). However, these prices are often observed just after harvest and are likely lower than the value that would have been observed later in the year. An alternative specification could value own consumption at the retail price of food purchases, which would result in a higher share of household income derived from agricultural production. However, we don't expect this variation would affect the broad lessons gleaned from our analysis.

4 In Tanzania, different government offices and ministries apply definitions of the term “urban” that differ mainly in the spatial unit of analysis (Muzzini and Lindeboom, 2008).

5 WorldPop refers to the most recent census data in each country, at the most granular level at which population numbers are reported, and then applies machine learning to relate population density values to other high-resolution data (e.g., land cover, telecommunications, and satellite data) in order to disaggregate population counts to smaller grids. The mapping approach is Random Forest-based dasymetric redistribution (WorldPop, n.d.).

6 In a robustness check, we have alternately followed the computation of Galdo et al. (2018), inserting Tanzania's GDP per capita to arrive at a digital number threshold of 14, and inserting Nigeria's GDP to arrive at a threshold of 22. The results of our analysis shift in predictable ways but are generally consistent.

7 In Figure A1 in the appendix, we explore the implication of applying different thresholds to these continuous variables. Using a lower population density threshold would have naturally produced a greater estimated urban population share, and this is 6 percentage points higher when the threshold is halved to 250 persons/km^2^.

8 It should be noted that Africapolis reports its own estimates of the urbanization level in each country, based on the population numbers ascribed to each urban polygon and an estimate of the country's total population. For Tanzania, this value is higher (at 38% (OECD/SWAC, 2020)) than what we calculate by overlaying the polygons on the Tanzania LSMS-ISA dataset. Africapolis refers to census numbers for the smallest geographic unit for which such numbers are available to estimate the urban population. However, it is not entirely clear how Africapolis ascribes these settlement size numbers to the Africapolis-generated polygons when the urban outline used for administrative purposes differs from that used by Africapolis. The current exercise, in which population numbers are drawn from a nationally representative, geographically explicit household survey, produces an estimate of a smaller number of people residing within the Africapolis urban areas.

9 This value is lower than the official estimate of urbanization in Nigeria, which stands at approximately 50% (Reed and Mberu, 2014). Nevertheless, the LSMS-ISA survey is designed to be representative of the rural and urban populations in Nigeria. Reed and Mberu (2014) elsewhere estimate the urbanization level in 2008 to be 36% which was 14 percentage points lower than the UN estimate, underscoring how difficult it is to estimate urbanization levels in Nigeria.

10 As in Tanzania, Africapolis estimates an urbanization level for Nigeria (at 51% (OECD/SWAC, 2020)) that is higher than we have calculated when overlaying the Africapolis urban polygons on the Nigeria LSMS-ISA. The reason for this divergence is not obvious, although we note that the population numbers used in Nigeria are from the 2006 census, for which numbers were reported only at the level of Local Government Areas (LGAs). At the time, there were just 774 LGAs in Nigeria. Given the size of Nigeria's population, this coarse breakdown may result in imprecision in the Africapolis estimates with respect to the population numbers ascribed to each urban polygon.

11 As noted by Rizzo et al. (2015), the distinction between income from wages and self-employment is ambiguous in labor surveys in Tanzania. However, this should not meaningfully affect the focus of our analysis, which is the share of income derived from agricultural sources.
